# Fix the Heart, Damage the Gut: A Case Report and Literature Review of Ablation-Induced Gastroparesis

**DOI:** 10.7759/cureus.23946

**Published:** 2022-04-08

**Authors:** Mrhaf Alsamman, Bilal Ashraf, Bernard Dankyi, Niti Aggarwal

**Affiliations:** 1 Internal Medicine, Health Corporation of America-University of Central Florida (HCA-UCF) Consortium, Ocala, USA

**Keywords:** cardiology, vagus nerve, cardiac ablation, atrial fibrillation, gastroparesis

## Abstract

Catheter ablation is a common procedure performed in patients with atrial fibrillation. While some commonly known complications include perforation, thromboembolism, pericardial effusion, and cardiac tamponade, gastroparesis is a less reported post-procedural complication. We present a case of a 66-year-old female admitted with intractable nausea and vomiting six weeks post-ablation. After ruling out the common causes of gastroparesis, a gastric emptying study was done, which confirmed the diagnosis of gastroparesis. Physicians should have a high degree of suspicion for vagus nerve damage in post-ablation patients that presents with unexplained persistent gastrointestinal (GI) symptoms to facilitate a better outcome.

## Introduction

Atrial fibrillation is a very common arrhythmia seen in the elderly population. Catheter ablation treatment is considered in patients with persistent atrial fibrillation despite medical therapy. Due to the proximity of the esophagus and left atrial posterior wall, damage to the vagal nerve branches during catheter ablation leading to gastroparesis has been described in the literature, but not yet confirmed [1]. Gastroparesis is defined as delayed gastric emptying without mechanical obstruction. Some common causes of gastroparesis include diabetes, amyloidosis, pancreatic adenocarcinoma, and mesenteric vascular insufficiency [2]. Here, we present a case of a 66-year-old female who presented with intractable nausea and vomiting six weeks post-ablation.

## Case presentation

**Figure 1:**
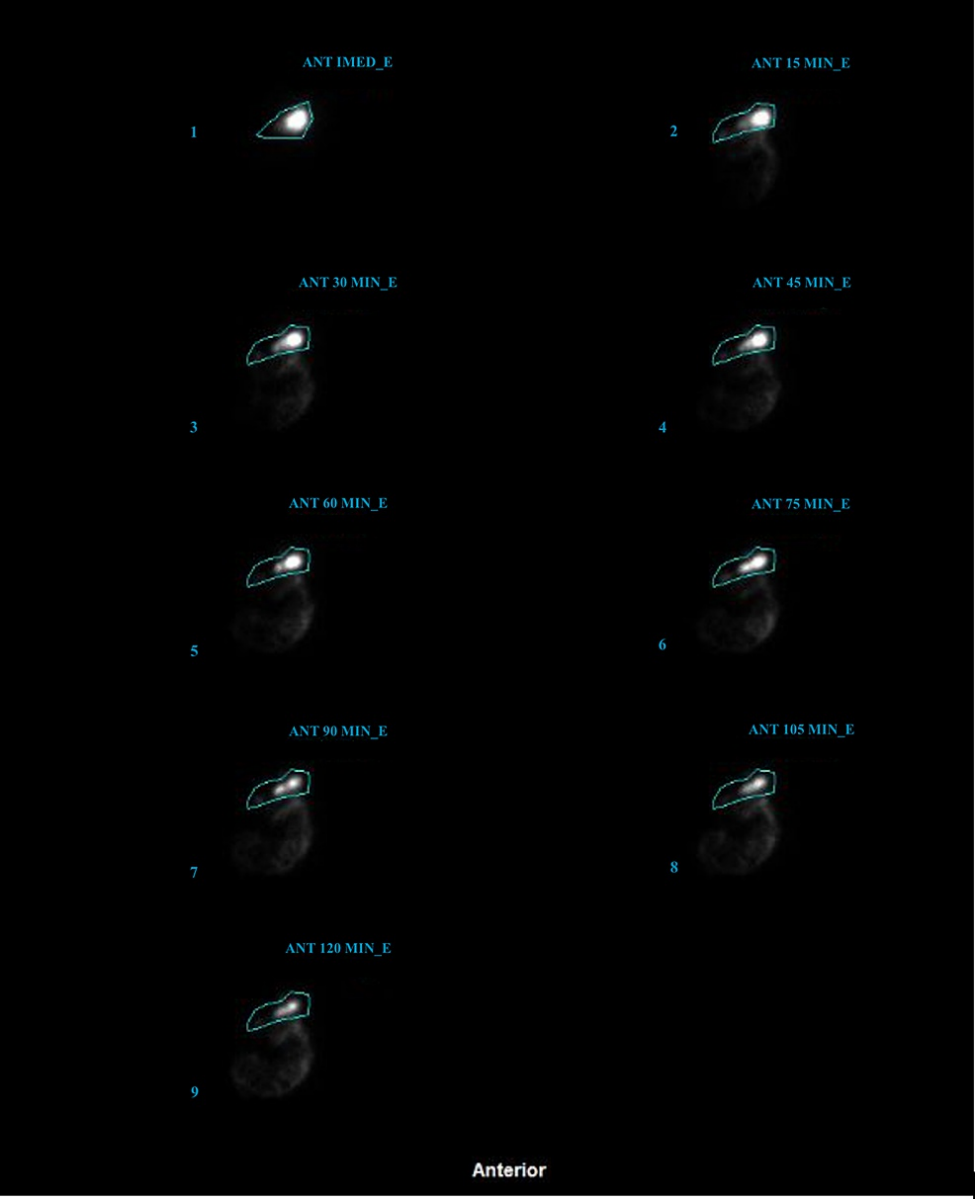
Gastric emptying scan reveals delayed gastric emptying time; contrast remained after 120 minutes, suggesting severe gastric hypomotility

**Figure 2:**
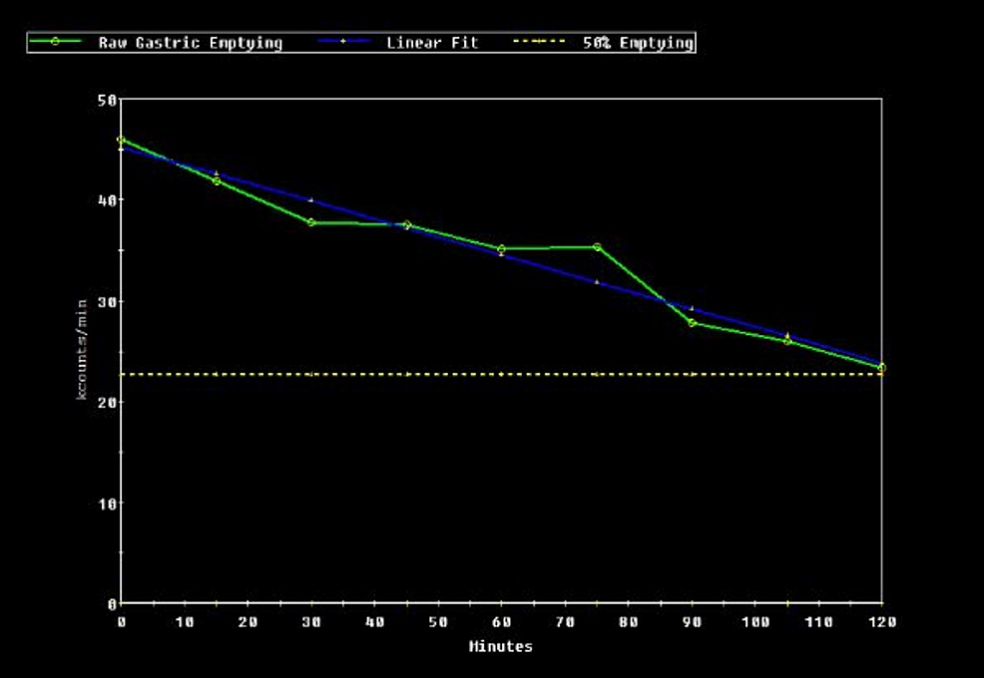
Gastric emptying graph demonstrating more than 50% contrast retaining after 120 minutes

A 66-year-old female presented to our facility with persistent nausea and vomiting for six weeks after having cryoablation for atrial fibrillation. The patient described an average of four vomiting episodes daily and associated 30-pound weight loss. The patient tried ondansetron and promethazine with no relief of her symptoms. On admission, laboratory results were significant for potassium of 3.0 mmol/L, total bilirubin of 2.1 mg/dL, AST of 56 u/L, ALT of 51 u/L, AM cortisol of 12.50 mcg/dL, negative hepatitis panel, ceruloplasmin of 31.1 mg/dL (normal range: 19-39 mg/dL), anti-mitochondrial antibody of 0.47 (normal range: 0-0.90), hemoglobin A1c of 4.8%, and negative COVID-19 PCR. Computed tomography (CT) of the abdomen with contrast, magnetic resonance imaging (MRI) of the brain with contrast, and liver ultrasound were unremarkable. The patient was started on metoclopramide 10 mg four times daily for presumed gastroparesis with significant improvement of her symptoms. The patient was able to tolerate the diet and was discharged. However, the patient returned after four weeks due to persistent nausea and vomiting despite metoclopramide compliance. A gastric emptying study was done, which confirmed gastroparesis demonstrating more than 50% contrast retaining after 120 minutes (Figures 1, 2). The patient was started on erythromycin, metoclopramide, and as-needed promethazine with improvement in her symptoms. The patient followed outpatient with a gastroenterologist for pyloric botulinum toxin injection.

## Discussion

The association between catheter ablation and the development of gastrointestinal (GI) dysmotility afterward has been reported in the literature; however, no definite mechanism of damage has been associated. Symptoms typically occur within 24 hours post-ablation and may persist for several months after that. In one study with 27 participants, 74% of the patients experienced dysmotility 24 hours after the procedure, and after three months, 33% of the patients had persisting symptoms such as nausea, bloating, and vomiting. All patients underwent gastric emptying study, esophageal manometry, and sham-feeding test before and after ablation [3]. The study reported that at six months post-procedure, the symptoms had resolved for almost all patients. In another study that looked at 100 patients who underwent ablation after atrial fibrillation, 40% of the patients continued to experience gastrointestinal (GI) symptoms including nausea, bloating, vomiting, feeling full, diarrhea, trouble swallowing, and constipation [4]. Among these patients, all symptoms had resolved by three months post-procedure, except for trouble swallowing, bloating, and early satiety or feeling full. These symptoms had also resolved by six months. One study that seems to contradict the high frequency of GI dysfunction and sampled 257 consecutive patients at a facility after ablation found symptoms in only five of the patients [5]. These patients mainly experienced symptoms of gastric hypomotility and acalculous cholecystitis. The studies conclude that GI disturbances are common post-procedure, but if symptoms persist, vagus nerve injury causing gastroparesis should be considered as a differential.

Vagus nerve damage seems to occur in patients who had multiple ablation procedures, and although there is no consensus on what causes the GI symptoms in these patients, it is widely believed in many studies that these symptoms are caused by thermal injury to the periesophageal plexus [5]. Some studies have postulated that patients with low BMI are likely to suffer vagus nerve injury and consequently GI dysfunction [6]. Other risk factors that have been observed include preexisting diabetes mellitus [4]. As of now, there are no known methods of reducing vagus nerve damage in patients who undergo catheter ablation after atrial fibrillation.

When a previously asymptomatic patient develops gastroparesis-like symptoms after catheter ablation, there is a high index of suspicion pointing toward damage to the paraesophageal vagus nervous plexus that controls gastric peristalsis, motility, and pyloric sphincter. However, other relatively common causes of gastroparesis and gastric dysmotility must be ruled out before making the diagnosis. The patient should be evaluated for diabetes, thyroid disease, GERD, recent viral illness, and other disorders such as sarcoidosis, scleroderma, systemic lupus erythematosus (SLE), and amyloidosis. A detailed history is beneficial to narrow down the possible etiologies causing the symptoms. A detailed screening for drug use and a thorough medication review can sometimes help identify the culprit as narcotics and anticholinergic medications can mimic gastroparesis and marijuana use can cause cannabis hyperemesis syndrome [7]. Esophagogastroduodenoscopy should be performed to rule out any mechanical obstruction. In the absence of any mechanical obstruction and any other known cause contributing to the patient’s symptoms, a gastric emptying study showing delayed emptying of the stomach confirms the diagnosis of gastroparesis.

Patients should be encouraged to take a small, frequent, low-fat, and low-fiber diet. Prokinetic medications such as erythromycin, metoclopramide, and mosapride citrate can also help in improving gastric motility, and symptomatic treatment with antiemetics is helpful. Enteral feeding and placement of percutaneous feeding tubes should be individualized based on patients’ nutritional status and the severity of symptomatology. Symptoms usually resolve spontaneously in three to six months. Patients with persistent symptoms may require botulinum toxin to relax the pyloric sphincter [8], endoscopic pyloroplasty, and surgical gastric pacemaker implantation [9].

## Conclusions

Although no definite mechanism of damage has been identified between catheter ablation and the development of gastrointestinal symptoms, physicians should have a high degree of suspicion for vagus nerve damage in post-ablation patients who present with unexplained persistent gastrointestinal symptoms to facilitate a better outcome.
